# Gout prevalence and management strategies among patients with moderate to advanced chronic kidney disease

**DOI:** 10.1590/2175-8239-JBN-2025-0282en

**Published:** 2026-04-17

**Authors:** Dhajanae Sylvertooth, Brian Bieber, Roberto Pecoits-Filho, Brad Marder, Brian LaMoreaux, Angelo Karaboyas

**Affiliations:** 1 Arbor Research Collaborative for Health, Ann Arbor, USA.; 2 Amgen, Thousand Oaks, USA.

**Keywords:** Gout, Hyperuricemia, Allopurinol, Renal Insufficiency, Chronic

## Abstract

**Introduction:**

Gout commonly coexists with chronic kidney disease (CKD), with prevalence increasing as kidney function declines. Despite its burden, data on monitoring and treatment practices in CKD are limited. This study describes the prevalence, characteristics, and management of gout in non-dialysis CKD patients.

**Methods:**

Cross-sectional data were analyzed from 3,524 stages 3a–5 CKD patients in the Chronic Kidney Disease Outcomes and Practice Patterns Study (CKDopps) in Brazil (n = 942) and the United States (n = 2,582) at enrollment (2013–2022). History of gout was extracted from medical records.

**Results:**

Gout prevalence was 18.7% overall—20.5% in the US and 13.9% in Brazil—higher in stages 4–5 vs. 3a–3b CKD. Allopurinol was most used (Brazil 75%; US 62%). Colchicine (13%) and febuxostat (8%) were reported in the US but rarely in Brazil. Uric acid was measured in 70% of Brazilian vs. 33% of US gout patients.

**Conclusions:**

Gout is common in CKD, with notable cross-country differences in monitoring and treatment.

## Introduction

Hyperuricemia is frequently observed in patients with chronic kidney disease (CKD) and becomes more prevalent and severe as kidney function declines. A strong link exists between uric acid concentrations and gout incidence, and urate-lowering treatment (ULT) is a central treatment for individuals exhibiting gout symptoms in both the general population and those with CKD. Despite the heightened prevalence of gout in CKD patients compared to the general population, recognition of gout and practice patterns in the CKD population warrant further investigation^1,2,3^.

Considering gout as a crucial indicator of hyperuricemia-induced inflammation, identifying the prevalence and characteristics of the affected individuals and practice patterns in the management of gout (including no treatment) are important to deepen the understanding of the hyperuricemia–inflammation–outcomes pathway in the CKD population. This multi-country study describes the prevalence, patient characteristics, and variations in the medical management of gout in a cohort of non-dialysis patients with moderate to advanced CKD across different countries.

## Methods

The Chronic Kidney Disease Outcomes and Practice Patterns Study (CKDopps) is a prospective cohort study, ongoing since 2013 in the US, Germany, France, and Brazil, designed to describe and evaluate variation in CKD practices and outcomes in nephrologist-led CKD clinics. Participating centers were sequentially (US, Germany) or randomly (Brazil, France) selected from stratified national samples of nephrologist-run CKD clinics, with a goal enrollment of 60 patients per unit for manual data collection and no limit for clinics participating via EHR extraction. The targeted ratio of enrolled patients with eGFR < 30 in relation to those with 30–59 mL/min/1.73 m^2^ was 2:1 in Brazil and the US; 3:1 in Germany; and 1:1 in France^4^.

This analysis includes 3,524 non-dialysis patients with stages 3a-5 CKD enrolled in Brazil (n = 942) and the United States (n = 2,582), in whom a history of gout was extracted from medical records. These medical records consist of comorbidities that were captured by study coordinators using standardized medical questionnaires across all countries, including gout status (yes or no) for each patient. This approach reduces misclassification that would occur if defined by the presence (vs. absence) of diagnostic codes. Patient characteristics, gout treatment, and uric acid measurement practices were summarized by gout diagnosis and country using cross-sectional data collected at study enrollment (2013–2022). The analyses performed were exclusively descriptive, and inferential results are not reported in this study.

## Results

Prevalence of gout was 18.7% overall – 20.5% in the US vs. 13.9% in Brazil – and was higher in stages 4–5 vs. stages 3a–3b CKD in both countries. Patients with (vs. without) a history of gout were more likely to be male (66% vs. 49%) and more likely to have a history of cardiovascular comorbidities, including congestive heart failure (US only), coronary artery disease, and cerebrovascular disease. Body mass index levels were similar regardless of gout status (Table 1).

**Table 1:** Characteristics of non-dialysis CKD patients with and without a history of gout

Baseline Characteristics	Brazil		United States	
	Gout = no n = 811 (86.1%)	Gout = yes n = 131 (13.9%)	Gout = no n = 2053 (79.5%)	Gout = yes n = 529 (20.5%)
Demographics				
Age, years	64.9 (14.8)	68.4 (12.4)	68.7 (13.0)	70.6 (11.7)
Male, %	50%	76%	48%	64%
Body mass index, kg/m^2^	27.7 (5.4)	28.2 (4.8)	31.8 (7.8)	32.6 (7.5)
eGFR	26.0 (11.7)	25.6 (11.1)	26.3 (10.8)	25.0 (9.7)
Comorbidities				
Diabetes	48%	34%	53%	52%
Coronary artery disease	20%	30%	27%	41%
Other cardiovascular disease	12%	21%	20%	30%
Congestive heart failure	16%	15%	15%	25%
Cerebrovascular disease	10%	12%	10%	12%
Cancer	9%	8%	19%	17%
Osteoporosis	8%	13%	7%	8%
Gout Medications				
Allopurinol	34%	75%	12%	62%
Colchicine	0.2%	5%	2%	13%
Febuxostat	0%	0%	0.8%	8%
Probenecid	0%	0%	0.1%	0.2%
Lab/biometric markers				
Uric Acid measured	48%	70%	16%	33%
Uric Acid, mg/dL	6.9 (1.8)	7.0 (2.3)	7.3 (2.0)	6.7 (2.1)
PTH, pg/mL	97 [54,174]	78 [47,175]	99 [62,164]	107 [70,170]
Phosphorus, mg/dL	4.2 (1.0)	4.1 (1.1)	3.9 (1.0)	3.9 (0.9)
Total Calcium, mg/dL	9.3 (0.8)	9.5 (0.7)	9.2 (0.6)	9.3 (0.6)
Serum Albumin, g/dL	3.9 (0.6)	4.2 (0.5)	3.8 (0.5)	3.8 (0.6)
Hemoglobin, g/dL	12.0 (1.9)	12.2 (1.9)	11.8 (1.9)	11.8 (1.9)
HbA1C, %	6.9 (1.5)	6.9 (1.6)	7.1 (1.6)	6.4 (1.2)
Total Cholesterol, mg/dL	177.2 (49.9)	180.2 (47.5)	169.9 (47.5)	169.3 (47.1)
Ferritin, ng/ml	143 [68,262]	171 [99,395]	144 [62,292]	183 [80,361]

Note – Mean ± standard deviation, median [interquartile range], or % shown.

Among patients with gout, allopurinol was the most commonly used treatment in both Brazil (75%) and the US (62%). Colchicine (13%) and febuxostat (8%) were also used in the US, but rarely in Brazil (≤ 5%). Mean uric acid among patients with gout was 7.0 mg/dL in Brazil vs. 6.7 mg/dL in the US; however, only 33% of US gout patients had a uric acid measurement recorded, compared to 70% in Brazil.

## Discussion

Prior research demonstrated that gout is underreported and potentially undertreated among North American patients with kidney failure on hemodialysis or peritoneal dialysis, with a prevalence of 13.9% in the dialysis setting^5^. In this study, we found that gout affects a similar proportion (18.7%) of the non-dialysis CKD population in Brazil and the United States, using the same method of capture as in Guedes et al. Prevalence of gout was higher for patients in the US compared to those in Brazil. The total prevalence of gout in this study is comparable to prior studies in other countries reporting a gout prevalence of 24.3% within the non-dialysis CKD population in Germany^6^ and 16.6% in the Republic of Ireland health system study in the CKD setting^7^.

Further, our results suggest potentially different management and monitoring strategies between the US and Brazil. Allopurinol emerged as the most commonly prescribed medication for gout in both countries under study. Interestingly, a significant observation was made: one-third of the patients in Brazil, who had no prior history of gout, were prescribed allopurinol. This contrasts with the United States, where only 12% of such patients received the medication. This disparity may imply that Brazilian nephrologists are leveraging allopurinol not just for gout but also for its broader therapeutic benefits. It is plausible that allopurinol is being prescribed to mitigate the risk of cardiovascular disease and progression of kidney diseases, addressing asymptomatic hyperuricemia. These insights suggest a more expansive approach to the use of allopurinol in Brazil, reflecting its potential utility in managing conditions beyond traditional gout treatment. Colchicine and febuxostat were rarely used in Brazil but were more common in the US. Additionally, US practices were less likely to measure uric acid levels, even among patients with a documented history of gout. These practice differences are consistent with a greater focus on hyperuricemia in Brazilian CKD practice and with efforts to measure and control uric acid levels beyond the context of gout.

Overall, this study highlights important variation in the diagnosis and treatment of gout across countries by exploring the characteristics and management of non-dialysis CKD patients. International variability in the management of gout and uric acid may reflect a lack of evidence to support clear guidelines or local preferences/availability of therapies. Further examination of the effectiveness of gout diagnosis and treatment practices between countries may provide an important opportunity to improve patient outcomes.

## Data Availability

The data that support the findings of this study are available from Arbor Research Collaborative for Health, but restrictions apply to the availability of these data. Data are available upon reasonable request and with permission from the institution.
