# Supplementary material for: Gout prevalence and management strategies among patients with moderate to advanced chronic kidney disease
Source: J Bras Nefrol. 2026 Apr 17;48(2):e20250282. doi: 10.1590/2175-8239-JBN-2025-0282en (PMC13120825; doi:10.1590/2175-8239-JBN-2025-0282en)
Supplement: Translation

# Translation: Prevalência de gota e estratégias de manejo em pacientes com doença renal crônica moderada a avançada

**DOI:** 10.1590/2175-8239-JBN-2025-0282pt

**Authors:** Dhajanae Sylvertooth, Brian Bieber, Roberto Pecoits-Filho, Brad Marder, Brian LaMoreaux, Angelo Karaboyas

**Affiliations:** 1 Arbor Research Collaborative for Health, Ann Arbor, USA.; 2 Amgen, Thousand Oaks, USA.

**Keywords:** Gota, Hiperuricemia, Alopurinol, Insuficiência Renal Crônica

## Abstract

**Introdução:**

A gota comumente coexiste com a doença renal crônica (DRC), com aumento da prevalência à medida que a função renal declina. Apesar de seu ônus, há poucos dados sobre as práticas de monitoramento e tratamento na DRC. Este estudo descreve a prevalência, as características e o manejo da gota em pacientes com DRC não dialíticos.

**Métodos:**

Foram analisados dados transversais de 3.524 pacientes com DRC estágios 3a–5 no *Chronic Kidney Disease Outcomes and Practice Patterns Study* (CKDopps), no Brasil (n = 942) e nos Estados Unidos (n = 2.582), no momento da inclusão (2013–2022). O histórico de gota foi extraído dos prontuários médicos.

**Resultados:**

A prevalência de gota foi de 18,7% no total — 20,5% nos EUA e 13,9% no Brasil — sendo maior nos estágios 4–5 quando comparada aos estágios 3a–3b da DRC. O alopurinol foi o medicamento mais utilizado (Brasil 75%; EUA 62%). Colchicina (13%) e febuxostato (8%) foram relatados nos EUA, mas raramente no Brasil. O ácido úrico foi dosado em 70% dos pacientes com gota no Brasil *versus* 33% nos EUA.

**Conclusões:**

A gota é comum na DRC, com diferenças marcantes entre os países quanto ao monitoramento e ao tratamento.

## Introdução

A hiperuricemia é frequentemente observada em pacientes com doença renal crônica (DRC) e torna-se mais prevalente e grave à medida que a função renal declina. Existe uma forte associação entre as concentrações de ácido úrico e a incidência de gota, e o tratamento para redução do ácido úrico (TRAU) constitui um pilar central no manejo de indivíduos com sintomas de gota, tanto na população geral quanto naqueles com DRC. Apesar da maior prevalência de gota em pacientes com DRC em comparação com a população geral, o reconhecimento dessa condição e os padrões de prática clínica nessa população ainda requerem investigação adicional^1,2,3^.

Considerando a gota como um importante indicador de inflamação induzida pela hiperuricemia, a identificação da prevalência, das características dos indivíduos afetados e dos padrões de prática no manejo da doença (incluindo a ausência de tratamento) é fundamental para aprofundar a compreensão da via hiperuricemia–inflamação–desfechos na população com DRC. Este estudo multicêntrico descreve a prevalência, as características dos pacientes e as variações no manejo médico da gota em uma coorte de pacientes não dialíticos com DRC moderada a avançada em diferentes países.

## Métodos

O *Chronic Kidney Disease Outcomes and Practice Patterns Study* (CKDopps) é um estudo de coorte prospectivo, em andamento desde 2013 nos Estados Unidos, Alemanha, França e Brasil, concebido para descrever e avaliar a variação nas práticas e nos desfechos da DRC em clínicas lideradas por nefrologistas especializadas no manejo dessa condição. As unidades participantes foram selecionadas de forma sequencial (EUA, Alemanha) ou aleatória (Brasil, França) a partir de amostras nacionais estratificadas de clínicas gerenciadas por nefrologistas, com o objetivo de incluir até 60 pacientes por centro para coleta manual de dados, sem limite para as clínicas participantes por meio de extração de dados de prontuários eletrônicos (EHR). A razão alvo de pacientes incluídos com eGFR <30 em relação àqueles com 30–59 mL/min/1,73 m^2^ foi de 2:1 no Brasil e nos EUA; 3:1 na Alemanha; e 1:1 na França^4^.

Esta análise inclui 3.524 pacientes não dialíticos com DRC estágios 3a–5 provenientes do Brasil (n = 942) e dos Estados Unidos (n = 2.582), nos quais o histórico de gota foi extraído dos prontuários médicos. Esses prontuários consistem em comorbidades registradas pelos coordenadores do estudo por meio de questionários médicos padronizados em todos os países, incluindo o *status* da gota (sim ou não) para cada paciente. Essa abordagem reduz a classificação incorreta que ocorreria caso a definição fosse baseada na presença (*versus* ausência) de códigos diagnósticos. As características dos pacientes, o tratamento da gota e as práticas de mensuração do ácido úrico foram resumidos de acordo com o diagnóstico de gota e o país, utilizando dados transversais coletados no momento da inclusão no estudo (2013–2022). As análises realizadas são exclusivamente descritivas, e resultados inferenciais não são apresentados neste estudo.

## Resultados

A prevalência de gota foi de 18,7% no total — 20,5% nos EUA *versus* 13,9% no Brasil — e foi maior nos estágios 4–5 em comparação com os estágios 3a–3b da DRC em ambos os países. Pacientes com histórico de gota (*vs.* sem histórico) eram mais propensos a serem do sexo masculino (66% *vs.* 49%) e apresentaram maior probabilidade de histórico de comorbidades cardiovasculares, incluindo insuficiência cardíaca congestiva (apenas nos EUA), doença arterial coronariana e doença cerebrovascular. Os níveis de índice de massa corporal foram semelhantes independentemente do *status* de gota (Tabela 1).

**Tabela 1:** Características de pacientes com DRC não dialítica com e sem histórico de gota

Características basais	Brasil		Estados Unidos	
	Gota = não n = 811 (86,1%)	Gota = sim n = 131 (13,9%)	Gota = não n = 2053 (79,5%)	Gota = sim n = 529 (20,5%)
Demografia				
Idade, anos	64,9 (14,8)	68,4 (12,4)	68,7 (13,0)	70,6 (11,7)
Sexo masculino, %	50%	76%	48%	64%
Índice de massa corporal, kg/m^2^	27,7 (5,4)	28,2 (4,8)	31,8 (7,8)	32,6 (7,5)
TFGe	26,0 (11,7)	25,6 (11,1)	26,3 (10,8)	25,0 (9,7)
Comorbidades				
Diabetes	48%	34%	53%	52%
Doença arterial coronariana	20%	30%	27%	41%
Outras doenças cardiovasculares	12%	21%	20%	30%
Insuficiência cardíaca congestiva	16%	15%	15%	25%
Doença cerebrovascular	10%	12%	10%	12%
Câncer	9%	8%	19%	17%
Osteoporose	8%	13%	7%	8%
Medicações para gota				
Alopurinol	34%	75%	12%	62%
Colchicina	0,2%	5%	2%	13%
Febuxostate	0%	0%	0,8%	8%
Probenecida	0%	0%	0,1%	0,2%
Marcadores laboratoriais/biométricos				
Ácido úrico medido	48%	70%	16%	33%
Ácido úrico, mg/dL	6,9 (1,8)	7,0 (2,3)	7,3 (2,0)	6,7 (2,1)
PTH, pg/mL	97 [54.174]	78 [47.175]	99 [62.164]	107 [70.170]
Fósforo, mg/dL	4,2 (1,0)	4,1 (1,1)	3,9 (1,0)	3,9 (0,9)
Cálcio total, mg/dL	9,3 (0,8)	9,5 (0,7)	9,2 (0,6)	9,3 (0,6)
Albumina sérica, g/dL	3,9 (0,6)	4,2 (0,5)	3,8 (0,5)	3,8 (0,6)
Hemoglobina, g/dL	12,0 (1,9)	12,2 (1,9)	11,8 (1,9)	11,8 (1,9)
HbA1C, %	6,9 (1,5)	6,9 (1,6)	7,1 (1,6)	6,4 (1,2)
Colesterol total, mg/dL	177,2 (49,9)	180,2 (47,5)	169,9 (47,5)	169,3 (47,1)
Ferritina, ng/mL	143 [68.262]	171 [99.395]	144 [62.292]	183 [80.361]

Nota – Média ± desvio padrão, mediana [intervalo interquartil] ou % apresentados.

Entre os pacientes com gota, o alopurinol foi o tratamento mais utilizado tanto no Brasil (75%) quanto nos EUA (62%). Colchicina (13%) e febuxostato (8%) também foram usados nos EUA, mas raramente no Brasil (≤ 5%). A média de ácido úrico entre pacientes com gota foi de 7,0 mg/dL no Brasil *versus* 6,7 mg/dL nos EUA; entretanto, apenas 33% dos pacientes com gota nos EUA tinham registro de medição de ácido úrico, comparado a 70% no Brasil.

## Discussão

Pesquisas anteriores demonstraram que a gota é subnotificada e potencialmente subtratada entre pacientes norte-americanos com insuficiência renal em hemodiálise ou diálise peritoneal, com uma prevalência de 13,9% nesse contexto^5^. Neste estudo, observamos que a gota afeta uma proporção semelhante (18,7%) da população com DRC não dialítica no Brasil e nos Estados Unidos, utilizando o mesmo método de registro utilizado em Guedes et al. A prevalência de gota foi maior entre os pacientes nos EUA em comparação com o Brasil. A prevalência total de gota neste estudo é comparável à observada em estudos anteriores em outros países, que relataram uma prevalência de 24,3% na população com DRC não dialítica na Alemanha^6^ e de 16,6% no estudo do sistema de saúde da República da Irlanda no contexto da DRC^7^.

Além disso, nossos resultados sugerem possíveis diferenças nas estratégias de manejo e monitoramento entre os EUA e o Brasil. O alopurinol surgiu como o medicamento mais comumente prescrito para gota em ambos os países estudados. Curiosamente, uma observação significativa foi feita: um terço dos pacientes no Brasil, que não apresentavam histórico prévio de gota, receberam prescrição de alopurinol. Em contraste, nos Estados Unidos, apenas 12% desses pacientes receberam o medicamento. Essa disparidade pode indicar que os nefrologistas brasileiros estão utilizando o alopurinol não apenas para tratar a gota, mas também por seus benefícios terapêuticos mais amplos. É plausível que o alopurinol esteja sendo prescrito para reduzir o risco cardiovascular e a progressão das doenças renais, abordando a hiperuricemia assintomática. Esses achados sugerem uma abordagem mais abrangente para o uso do alopurinol no Brasil, refletindo seu potencial na gestão de condições além do tratamento tradicional da gota. Colchicina e febuxostato foram raramente utilizados no Brasil, mas foram mais comuns nos EUA. Além disso, as práticas nos EUA apresentaram menor frequência na medição dos níveis de ácido úrico, mesmo entre pacientes com histórico documentado de gota. Essas diferenças de prática são consistentes com um foco maior na hiperuricemia na prática da DRC no Brasil, incluindo esforços para mensurar e controlar os níveis de ácido úrico além do contexto da gota.

De modo geral, este estudo destaca uma variação importante no diagnóstico e no tratamento da gota entre países, ao explorar as características e o manejo de pacientes com DRC não dialítica. A variabilidade internacional no manejo da gota e do ácido úrico pode refletir a falta de evidências para embasar diretrizes claras ou preferências locais/disponibilidade de terapias. Uma análise mais aprofundada da eficácia das práticas de diagnóstico e tratamento da gota entre países pode oferecer uma oportunidade importante para melhorar os desfechos dos pacientes.

## Data Availability

Os dados que sustentam os achados deste estudo estão disponíveis no *Arbor Research Collaborative for Health*, mas há restrições quanto à disponibilidade desses dados. Os mesmos podem ser obtidos mediante solicitação razoável e com permissão da instituição.
